# Clinical, Imaging, and Pathological Suppression of Synovitis in Rheumatoid Arthritis: Is the Disease Curable?

**DOI:** 10.3389/fmed.2018.00140

**Published:** 2018-05-15

**Authors:** Serena Bugatti, Garifallia Sakellariou, Terenzj Luvaro, Maria Immacolata Greco, Antonio Manzo

**Affiliations:** Division of Rheumatology, Rheumatology and Translational Immunology Research Laboratories (LaRIT), IRCCS Policlinico San Matteo Foundation and University of Pavia, Pavia, Italy

**Keywords:** Rheumatoid arthritis, remission, drug-free remission, synovitis, ultrasonography

## Abstract

The management of patients with rheumatoid arthritis (RA) has witnessed a dramatic revolution in recent years, and disease remission has become an increasingly achievable outcome. Rheumatologists are now facing the urgent question of whether, once remission has been achieved and stably maintained, drugs can be tapered, and even discontinued. The concept of disease remission however encompasses progressive layers of complexity, all of which need to be disentangled before considering RA as a “curable” condition. As the synovial membrane represents the ultimate target of the pathological process of RA, a critical issue remains whether disease remission coincides with true suppression of inflammation and definitive tissue “healing.” In this short review, we will provide a critical summary of recent studies investigating the possibility of controlling RA synovitis at the clinical, imaging or pathological level. Potential advantages and limitations of these perspectives in the definition of remission are also discussed.

Disease remission has become an increasingly achievable outcome in patients with rheumatoid arthritis (RA). Modern trials in early RA report remission rates of up 50–60% with methotrexate (MTX) combined with glucocorticoids (1–3), and real-life data on early RA cohorts managed according to the principles of early diagnosis and goal-steered treatment strategies confirm remission in 30–50% of the cases at 1 year (4–6).

Stringent control of disease activity minimizes the risk of joint destruction, functional disability and overall mortality in patients with RA (7). Furthermore, aiming for remission undoubtedly represents the pre-requisite for an ambitious outcome in the modern management of the disease, that is tapering and eventual discontinuation of therapies. Accordingly, the latest American (8) and European (9) recommendations include cautious indications on de-escalation of biological (b) and conventional synthetic (cs) disease modifying anti-rheumatic drugs (DMARDs). However, discontinuation of therapies exposes to an increased risk of relapse in up 50% of the cases (10, 11). The identification of patients in whom de-escalation of DMARDs is more appropriate remains an area of uncertainty, but presence of “deep” remission, although not sufficient, appears reasonably associated with higher chances of maintaining remission once drugs have been stopped (10). In that sense, the persistence of “deep” remission off-therapies might truly coincide with definitive cure of RA.

At present, the definition of “deep” remission in RA remains uncertain. It is well established that clinical instruments detecting signs and symptoms of inflammation may allow the persistence of residual sub-clinical disease activity at imaging, and further analyses at the tissue level may reveal additional layers of complexity. In this short review, we will summarize recent data on the prevalence and significance of progressive layers of disease remission in RA, with focus on suppression of synovitis from a clinical, imaging, and pathologic perspective.

## Lessons from inflammatory bowel diseases

The complexity of the assessment of inflammation and disease activity, with the existence of multiple progressive layers, is well established in other immune-mediated diseases such as Crohn's disease (CD). Similarly to RA, clinical indexes such as the Crohn's disease activity index (CDAI) have been developed with the aim of making clinical assessment quantifiable and reproducible (12). However, clinical symptoms in CD are not very specific and hence do not always correlate with objective signs of disease activity (13). In particular, the CDAI cut-off of remission of <150 allows the persistence of some degree of endoscopic disease activity in about half of the patients (13). For a better definition of disease activity, clinical assessment might be usefully integrated with imaging techniques such as computed tomography- or magnetic resonance imaging (MRI)-enterography, which have been shown to correlate well with endoscopic findings and to be highly predictive for long-term outcomes (14). At present, the gold-standard for assessing disease activity in CD remains however endoscopy, and the ultimate therapeutic target is consistently recognized as mucosal healing (15). The definition of mucosal healing is still debated but, interestingly enough, the cut-off values for remission of the main endoscopic disease activity indexes all allow a certain degree of inflammation and/or damage to persist (16). Furthermore, persistent histological inflammation occurs in 25% of patients with clinically and endoscopically quiescent CD (17). This opens the question on whether more stringent definitions of remission should be pursued, and whether definitive histologic healing of intestinal inflammation might be obtained. From a pathophysiological perspective, mechanisms of mucosal restitution and repair have been characterized, and include suppression of inflammation and enhanced barrier function (18). These complex processes are controlled by regulatory growth factors, cytokines and bacterial products, which induce specific intracellular signaling cascades leading to activation of master transcription factors in epithelial cells (18, 19). Histological parametres of disease resolution include absence of epithelial damage, resolution of infiltration of the epitelium, and the lamina propria, absence of erosions/ulcers and granulomas (20). If achievable, “complete” remission would imply full concordance between clinical, endoscopic, and histological remission. Whether this ambitious outcome guarantees drug-free survival and abrogates long-term complications in patients with CD remains however to be demonstrated.

## Clinical remission

From a clinical perspective, suppression of disease activity in RA ideally implies the absence of any detectable swollen and tender joint for a prolonged period of time, as well as abrogation of any sign of systemic inflammation. In practice, there is no definitive answer on how often clinical remission could be achieved due to lack of standardization of several aspects including (i) the clinical index used to define remission; (ii) the duration of remission; (iii) whether remission is intended in course of treatment or rather after drug tapering/suspension.

It is well established that all the clinical indexes currently used to define disease remission may allow some degree of residual inflammation at the patient level. The loosest definition of remission is attributed to the 28-joint disease activity score (DAS28) criterion (21), although, when DAS28 remission is also sustained, only a minority of patients have clinically detectable joint involvement (22, 23). By definition, remission according to the simplified and the clinical disease activity index (SDAI, CDAI) allows a maximum of 2 clinically active joints (24), whilst Boolean remission is very close to full suppression of synovitis by allowing no more than 1 swollen and tender joint (25). More ambitious definitions require a tender joint count of 0, a swollen joint count of 0 and an erythrocyte sedimentation rate ≤ 10 mm/1 h (OMERACT 7) (26). In keeping with such progressive stringency, remission in early RA is quite common according to the DAS28 criterion, and relatively rarer for the SDAI and the Boolean cut-offs(27–30).

Unfortunately, clinical control of disease activity is not protective for disease recurrence over time. Irrespective of whether therapy remains unchanged, sustained remission is indeed much rarer than point remission in both clinical trials and routine care. In the treatment in the Rotterdam Early Arthritis Cohort (tREACH) trial, 53–59% of the patients were able to sustain DAS remission for two consecutive visits (31). Real-life data, however, consistently report much lower rates, irrespective of the remission criterion adopted. In the Nijmegen early RA inception cohort, DAS remission was sustained in 19% of the cases (32). Similarly, in the Canadian early ArThritis CoHort (CATCH) study, sustained SDAI remission was observed in 23% of the patients, and Boolean remission in <19% (33). Even in patients experiencing full suppression of synovitis (i.e., no swollen and tender joints), remission at follow-up is maintained only in a minority of the cases (34), so that no more than 10% of the patients in different cohorts experience disease recovery while treated(22, 34, 35).

When suppression of synovitis persists after drug discontinuation, the disease might be virtually considered as “cured.” Observational studies and controlled trials have shown that sustained drug-free remission is actually possible in RA, although its achievement appears restricted to a minority of the patients. The Dutch Behandel-Strategieën (BeSt) randomized controlled trial initially showed that, irrespective of the treatment arm, just 15% of the patients were in drug-free remission after 10 years (36). More recently, in the Induction therapy with MTX and Prednisone in Rheumatoid Or Very Early arthritic Disease (IMPROVED) study, approximately 20% of the patients were in drug-free remission after 2 years of remission-steered treatment (37). In the large observational inception cohorts from the Leiden Early Arthritis Clinic, The Netherlands, and the British Early Rheumatoid Arthritis Study, sustained DMARD-free remission, defined as a sustained absence of clinical synovitis for at least 1 year after cessation of DMARDs, was achieved in 9–15% of patients during follow up (38). Collectively, drug-free remission is more probable if DMARDs are commenced early, if remission is achieved early in course of disease and if treatment is steered at the target (39). Furthermore, irrespective of how deeply inflammation is controlled, autoantibody-positive patients less frequently sustain remission (10, 39), raising the possibility that “cure” of seropositive RA might require full recovery of immune abnormalities beyond suppression of disease activity.

In summary, evaluating remission exclusively from a clinical perspective (even through most stringent criteria) appears unsuitable to capture RA patho-biology in its full complexity, pointing at the existence of further stratification levels, and the requirement of complementary analytical tools (Table 1).

**Table 1:** Assessment of disease activity/remission according to current perspectives.

**Approach**	**Advantages**	**Limitations**
Clinical perspective	1- Offers a definition of remission according to validated thresholds	
	2- applicable in routine clinical practice	
		3- does not allow to measure sub-clinical inflammatory activity
		4- does not allow to measure synovial stroma pathology
	5- allows to measure disease activity according to a systemic perspective 6- allows to define remission stability in longitudinal terms based on serial assessments	
US and MRI perspective		1- does not (yet) offer a definition of remission according to validated thresholds
		2- requires equipment and experienced operators
	3- allows to measure sub-clinical inflammatory activity (according to surrogate markers)	
		4- does not allow to measure directly synovial stroma pathology
	5- allows to measure inflammatory activity according to a multi-site perspective (US)	
	6- allows to measure inflammation stability in longitudinal terms based on serial assessments	
Pathological perspective		1- does not (yet) offer a definition of remission according to validated thresholds
		2- limited applicability in routine clinical practice (requires *ad-hoc* facilities)
	3- allows to measure sub-clinical inflammatory activity (according to direct markers)	
	4- allows to measure synovial stroma pathology	
		5- does not allow to measure inflammatory activity according to a systemic or multi-site perspective
		6- does not (routinely) allow to measure inflammation stability in longitudinal terms based on serial assessments

## Imaging remission

According to this concept, in patients achieving clinical remission, imaging can capture both structural damage and inflammatory lesions. While structural damage could be the result of previous phases of disease activity, inflammatory lesions, which can be detected by modern approaches such as MRI and ultrasonography (US), may allow description of relevant signs of residual disease activity.

In particular, despite more selective criteria (notably SDAI and Boolean definitions of remission) are associated with lower prevalence of US abnormalities, both in cross-sectional (40) and longitudinal studies (41), US gray-scale (GS) synovitis has been reported in up to 79% of patients meeting Boolean remission and PD positivity in up to 32% (42). As GS abnormalities are frequently detected in healthy subjects, it can be questioned whether these reflect true disease activity, although PD is less commonly seen in healthy joints and might be a truthful indicator of residual inflammation. In keeping with this consideration, a study analysing 55 patients in DAS28 remission demonstrated that patients with residual PD had higher levels of angiogenetic biomarkers (43), supporting the concurrent validity also of subclinical PD. Moreover, synovial samples obtained from patients in DAS28 remission with residual PD showed significantly more vascularisation compared to those of patients without arthritis and a similar degree of macrophage infiltration compared to active RA (44). The persistence of subclinical synovitis in clinically silent joints seems to be a long-lasting phenomenon, which however might tend to attenuate over time, although only indirect evidence supports the latter hypothesis (45). Residual joint inflammation in patients in remission can be demonstrated also by other imaging techniques. An MRI study showed that residual synovitis and bone marrow edema (BME) persisted in 90 and 31% at the wrists (46). Similarly, a small pilot study using positron emission tomography (PET) with a specific technique tracing macrophages showed that, in a population of 25 patients in minimal disease activity (DAS < 1.6, no swollen or tender joints), 44% had enhanced tracer uptake in at least one joint at hand and wrists (47).

A number of US studies has underlined the relevance of the persistence of imaging-detectable inflammation over longitudinal outcomes. In many independent cohorts, in fact, the presence of PD synovitis has been shown to be an independent predictor of disease flare (48, 49), especially in patients with early disease, while this effect was less pronounced in long-standing disease (50). In this scenario, also tenosynovial PD has been related to self-perceived unstable remission (51). Interestingly, residual joint inflammation visible at PET was more frequent in patients with subsequent flare, while MRI was unable to detect differences among patient groups (47). Persistent inflammation at imaging may also explain the possible, albeit small, joint damage progression described in patients in clinical remission (52). In a population of patients in clinical remission as defined by the treating rheumatologist, a significant proportion of patients showed subclinical US and MRI activity (53), and PD and MRI synovitis were the only significant predictors of radiographic progression at follow-up in univariate analysis (54). Damage progression at MRI, which has a greater sensitivity to change compared to conventional radiography, has also been used as a measure of outcome in patients in clinical remission, in which a continuous structural deterioration has been shown. This continuous progression was associated with the persistence of synovial and tenosynovial inflammation (55).

Given the prognostic relevance of subclinical inflammation, interest has raised in using imaging remission as a therapeutic target. So far, two studies compared clinical and US treat-to-target strategies in patients with active RA, however both trials failed to detect significant differences between the two arms in medium-term outcomes (56, 57). A randomized controlled trial aiming at suppression of MRI BME has been completed, although results are still not available (NCT01656278). These trials enrolled patients with active disease, while strategic trials in which imaging is integrated in the clinical management of patients in clinical remission are still lacking. Nevertheless, these negative results highlight a gap in our knowledge about the relevance of imaging-detected inflammation. A part of this gap might be explained by the absence of consistent definitions of imaging-detected abnormalities. While thresholds of inflammatory activity which have no impact over relevant outcomes have been defined for MRI (58), US thresholds for GS and PD that reliably identify true disease activity and can distinguish healthy subjects from RA patients have yet to be individuated. In addition, the optimal US scanning protocol has not been clarified yet, with a great heterogeneity regarding the sites and the number of joints to be assessed among studies (59, 60).

Notwithstanding these limitations, the frequent detection of imaging inflammation in patients supposed to do well lends further support to the incapacity of any of the conventional definitions of remission to identify a full and complete control of the disease. Whilst imaging findings appear related to poorer outcomes, their full relevance and broad applicability has however to be better clarified through strategic trials and optimization of valid cut-offs able to translate the concept of remission in quantitative terms (Table 1).

## Pathological remission

Altogether, the above described findings indicate that clinical synovitis can be persistently suppressed in a small but significant proportion of RA patients (39). Although rarer, imaging signs of synovial inflammation may also disappear, particularly in joints with longer periods of clinical inactivity (45). This raises the question of whether the injured synovial tissue may ever return to normalcy in patients experiencing sustained and deep remission.

Synovial pathologic changes in course of RA encompass alterations of the stromal scaffold as well as infiltration by circulating leukocytes (61). Both the components are required to regress in order to define the synovium as “healed.” From a biological perspective, resolution of tissue inflammation is actively mediated by endogenous mediators such as autacoids, polypeptides, and proteins (62). *In vitro* studies and experimental arthritis models have shown that a number of pro-resolving factors are effective at inhibiting tissue infiltration and fibroblast pathologic behaviors. Galectins −1 and −9 shift T lymphocyte polarization toward type 2 helper and regulatory cells (63, 64), and induce apoptosis of fibroblast-like synoviocytes (FLS) (65). Melanocortins such as ACTH control cytokine release and macrophage phagocytosis and inhibit inflammation in experimental arthritis (66). Regulatory T cell production of adenosine, a nucleoside capable of reducing the accumulation and function of leukocytes, is significantly increased by MTX (67). Also, synovial macrophages, endothelial cells, and FLS express lipid pro-resolving mediators such as lipoxygenase, able to activate resolution circuits through cognate receptors (68). Recently, a novel cytokine pathway involved in resolution of inflammation, characterized by interleukin-9 production by type 2 innate lymphoid cells, has been described (69). In addition to the possible expression of pro-resolving mediators and cytokines, there is also experimental evidence indicating that cells and fluids can be actively drained from the inflamed synovium through the lymphatic vascular network (70, 71).

Altogether, these data indicate that, potentially, pro-resolving mechanisms might be operating in the inflamed synovium. However, the expression profiles of lipid mediators, proteins and their receptors, as well as lymphatic egress, have not been routinely measured across different phases of disease activity in patients with RA. As such, it is currently unknown whether disease remission coincides with the activation of pro-resolving pathways within the synovium.

Despite the mechanisms effectively operating in tissue healing in course of RA remain to be demonstrated, recent evidences are starting to suggest that, at least histologically, the infiltrating component of the synovial lesion may be significantly modulated in course of clinical remission. In RA patients with no evidence of swollen and tender joints but persistence of PD-positive synovitis, the density of infiltrating lymphocytes appears reduced compared to active RA (44). However, some degree of histological inflammation persists, event in joints with no signs of imaging activity (72). In particular, clinical and PD inactive joints have been shown to exhibit similar amounts of T and B lymphocytes as compared with joints from patients in low disease activity (72). Relevantly, the level of residual leukocyte infiltration, particularly of B cells, appears predictive of clinical flare in the short-term (44). Subclinical inflammation not even detected by imaging would thus underlie episodes of clinical recurrence.

Data on the possible reversal of stromal pathological changes are even more scarce. Although the density of hsp47+ FLS was significantly reduced in RA patients in remission compared to active RA (44), collagen deposition in the synovial lining and sublining was found to be increased in inactive joints (72). This latter finding may indirectly suggest that resolution of inflammation is histologically characterized by progressive fibrosis, and that suppression of clinical and imaging synovitis does not coincide with restoration of the normal synovial architecture. Full proof of this concept is lacking, but the persistence of GS synovitis in the vast majority of RA patients despite stringent clinical remission (42) lends support to histological findings. Interestingly, the observation that synovial hyperthrophy by US is associated with recurrence of disease in patients with psoriatic arthritis upon drug discontinuation (73) raises the possibility that tissue damage in arthritis cannot be effectively controlled, and that joints remain “primed” despite resolution of clinical, imaging, and histological inflammation.

In conclusion, despite the applicability of synovial tissue analysis in the assessment of remission may face practical limitations, mostly related to the polyarticular, and systemic nature of RA (74) (Table 1), the data summarized in this paragraph emphasize the existence of relevant pathologic aspects of the disease that may fail to translate into conventional clinical or imaging signs. Further advancements in the histological and molecular characterization of the synovium at sub-clinical and sub-inflammatory level, might thus turn out as relevant steps for full comprehension of remission and residual disease.

## Conclusions

**Figure 1:**
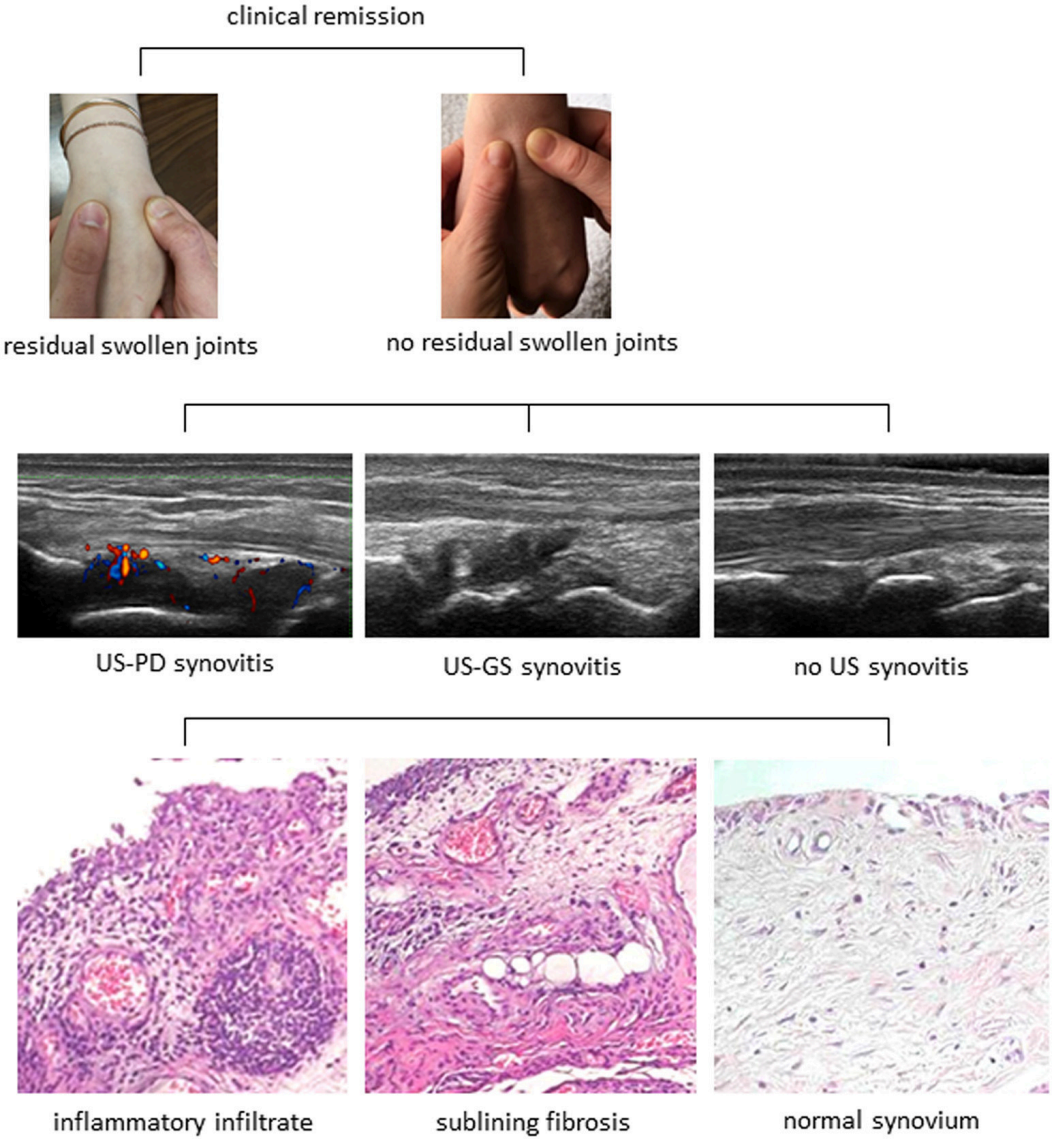
The spectrum of disease activity in rheumatoid arthritis. The progressive layers of disease activity in rheumatoid arthritis are shown. A clinically non-swollen joint may still exhibit signs of inflammation at ultrasonography (US), as either Power Doppler (PD) positive synovitis, or gray scale (GS) synovitis. Furthermore, both active and inactive joints at imaging can present variable degrees of histopathological inflammation with leukocyte infiltration of the synovial sublining and/or stromal alterations. Tissue healing with complete suppression of inflammation and restoration of the stromal architecture would coincide with “cure” of the disease.

The remarkable advancements in disease diagnosis and treatment over the past decade have dramatically improved the outcomes and expectations of patients with RA. Albeit numerically small, a proportion of patients may even experience sustained drug-free remission. Collectively, this opens the important question of whether synovitis can be fully and stably suppressed, with return to normalcy. Currently available data however demonstrate that only a minority of RA patients in remission have clinically inactive joints. Furthermore, despite the absence of clinical signs of inflammation, sensitive imaging techniques can still reveal a certain amount of subclinical disease activity (Figure 1). Also, recent data is starting to indicate that joint inactivity at imaging does not necessarily coincide with resolution of inflammation at the histological level (Figure 1). Finally, disappearance of the synovial inflammatory infiltrate wouldn't *per se* be synonymous of complete “healing,” as the stromal scaffold might remain “injured” and eventually more subjective to disease recurrence upon minor stimuli (Figure 1). More detailed understanding of the mechanisms of tissue inflammation and repair is thus needed before considering RA as a “curable” disease.

## Author contributions

SB, GS, TL, MG, and AM contributed to literature review and preparation of the manuscript.

### Conflict of interest statement

The authors declare that the research was conducted in the absence of any commercial or financial relationships that could be construed as a potential conflict of interest.
